# Alteration of metabolic profiles in *Lemna paucicostata* culture and enhanced production of GABA and ferulic acid by ethephon treatment

**DOI:** 10.1371/journal.pone.0231652

**Published:** 2020-04-16

**Authors:** EunBi Kim, Myeongsun Kim, Hyung-Kyoon Choi

**Affiliations:** College of Pharmacy, Chung-Ang University, Seoul, Republic of Korea; Rochester Institute of Technology, UNITED STATES

## Abstract

*Lemna* species have been used in the food, feed, and pharmaceutical industries, as they are inexpensive sources of proteins, starches, and fatty acids. In this study, we treated *L*. *paucicostata* with different concentrations (0.05, 0.1, 0.2, 0.5, or 1 mM) of ethephon. The total dry weight decreased in all ethephon-treated groups compared to the control group. We also investigated the alteration of metabolic profiles induced by ethephon treatment by using gas chromatography-mass spectrometry. This analysis identified 48 metabolites, and the relative levels of most of alcohols, amino acids, fatty acids, and phenols increased by the ethephon treatment, whereas levels of organic acids and sugars decreased. Among these, the highest production of γ-aminobutyric acid (GABA, 5.041 ± 1.373 mg/L) and ferulic acid (0.640 ± 0.071 mg/L) was observed in the 0.5 mM and the 0.2 mM ethephon treatment groups, respectively. These results could be useful for large-scale culture of *L*. *paucicostata* with enhanced GABA and ferulic acid content for utilization in the food, feed, cosmetic, and pharmaceutical industries.

## Introduction

*Lemna paucicostata*, also known as *Lemna aequinoctialis*, is one of the 37 known *Lemnaceae* species, which typically inhabits lakes as a floating plant [1]. *Lemnaceae* species have been used as animal and fish feed worldwide because it grows rapidly, it is easily harvested, and non-toxic [2]. *Lemnaceae* species containing a high concentration of inexpensive proteins, starches, and fatty acids are already used in the food industry, in soups, curries, and salads [3]. Additionally, they are also widely used traditionally as analgesics, anthelminthics, antiinflammatory medicines, and for the treatment of some throat and nose diseases in China, Russia, and some European countries [4].

Ethylene is a well-known plant hormone that regulates cell growth, reproduction, senescence, and various metabolites production in plants [5, 6]; interestingly, unlike any other plant hormone, ethylene is a gas. Previous studies have shown that 1-amino cyclopropane-1-carboxylic acid, a precursor of ethylene, reduced growth and fresh weight but increased the chlorophyll content of *Lemna minor* [7]. Furthermore, ethylene resulted in fast underwater elongation of *Oryza sativa* and *Rumex palustris* [8]. Although ethylene is unique and has a simple structure, it is difficult to apply it directly to plants because of its gaseous nature and low solubility. However, ethephon, a precursor of ethylene, is easily transformed to ethylene upon metabolism by plants, whether in solution with a weak acid or base [9]. Thus, various studies have used ethephon instead of ethylene. Ethephon is used not only as a growth regulator [10] but also as an inhibitor of shoot and root production [11, 12], a flowering and vegetation accelerator [13], a fruit aging inducer [14], a fruit color enhancer [15], a vermin exterminator [16], and a secondary metabolite promotor [17, 18].

Metabolomics is the comprehensive and quantitative analysis of metabolites obtained in biological samples [19]. With an increasingly wide application in recent years, metabolomics is a useful tool to observe the regulation of metabolic networks. Additionally, it is used to select metabolic biomarkers, elucidate plant responses to stress, identify bacteria, and evaluate human health [20, 21]. The adoption and development of metabolomics for plant studies make it possible to observe comprehensive metabolic networks and reactions against environmental stress. This is achieved by using vibrational spectroscopy, nuclear magnetic resonance, and mass spectrometry, including gas chromatography–mass spectrometry (GC-MS), liquid chromatography–mass spectrometry, capillary electrophoresis–mass spectrometry, or direct infusion mass spectrometry [22]. Owing to the fact that GC-MS has well-established methods and analysis pipelines, it has been widely adopted for the identification and quantification of plant metabolites [23, 24].

We hypothesized that exogenous ethephon modulate the growth and metabolite profile of *L*. *paucicostata* culture. We employed GC-MS to investigate the metabolic alteration in *L*. *paucicostata* cultures exposed to varying concentrations of ethephon. The main aims of this study were to investigate the effects of various concentrations of ethephon on the growth and metabolic profiles of *L*. *paucicostata* culture, and to suggest an optimal concentration of ethephon for enhancing production of valuable metabolites.

## Materials and methods

### Cultivation of *L*. *paucicostata*

*L*. *paucicostata* PC-10605 strain was obtained from the Korean Collection for Type Cultures (Biological Resource Center, Daejeon, Republic of Korea). Cultivation of *L*. *paucicostata* in solid media was conducted according the procedure described previously [25]. Sub-culturing of *L*. *paucicostata* was conducted every two weeks. For adaptation to liquid culture conditions, *L*. *paucicostata* was cultivated in one liter of medium for three days. After adaptation to liquid culture, 30 plants were placed in a 200 mL flask (Diamond, Republic of Korea) containing 100 mL of the culture medium for each group with varying concentrations of ethephon. The experiments were performed in three biological replications. Samples were harvested on day 35, freeze-dried (Bondiro, Ilshin Lab. Co., Seoul, Republic of Korea) for 48 h, and stored at −75°C until analysis.

### Growth measurement

Ethephon was dissolved in the *L*. *paucicostata* growth media to concentrations of 0.05, 0.1, 0.2, 0.5, or 1 mM. The total number of *L*. *paucicostata* fronds was counted every 7 d for 35 d (S1 Fig). For measurement of total dry weight (DW), harvested whole plants were washed with distilled water under vacuum filtration using Whatman filter paper (Whatman No. 2, Whatman, Maidstone, UK) and suction flask. Then, the plants were dried for 15 min, and moved to conical tubes. After freeze-drying, their total DW was measured.

### Comprehensive metabolite profiling by GC-MS

Samples were ground before performing gas chromatography-mass spectrometry (GC-MS) analysis; the extraction method was based on Kim et al. [26]. As an internal standard (IS), 10 μL of 2,000 mg/L myristic-*d*_*27*_ acid (Tokyo Chemical Industry Co., Japan) in pyridine was used. The GC-MS analysis conditions used were as previously reported after modification of oven temperature settings [23]. At the start of the experiment, the oven temperature was set to 60°C and programmed to increase to 140°C (at 5°C/min), then to 150°C (at 10°C/min and holding for 3 min), then to 180°C (at 3°C/min), then to 185°C (at 5°C/min and holding for 3 min), then to 200°C (at 3°C/ min), then to 225°C (at 5°C/min), then to 230°C (at 3°C/min), then to 280°C (at 5°C/min), and finally to 310°C (at 8°C/min), thus making the total runtime 59.42 min. GC-MS analyses were performed in triplicate.

Spectral data containing the retention time and mass to charge ratio (m/z) data of each peak were imported into Expressionist MSX software (version 2013.0.39, Genedata, Switzerland) and converted to .csv format (Excel Office 2016). The assignment of peaks obtained by GC-MS analyses was conducted by matching the spectral data of each peak from samples with spectral libraries of various databases. Peaks with a match > 70% were selected. The Golm Metabolome Database (gmd.mpimp-golm.mpg.de/), Human Metabolome Database (HMDB), and National Institute of Standards and Technology (NIST) were used for peak assignment. Normalization of each peak was performed by dividing the intensity of each peak by the intensity of the IS to show the relative levels of metabolites in each sample.

### Quantification of GABA and ferulic acid

Quantification of GABA, caffeic acid, and ferulic acid was accomplished using a calibration curve constructed for the GABA standard (0.3125, 0.625, 1.25, 2.5, 5, 10, 20, 40, and 80 mg/L), the caffeic acid standard (0.5, 1, 2, 4, 8, and 16 mg/L), and another curve for the ferulic acid standard (0.5, 1, 2, 4, 8, and 16 mg/L). The calibration curves were obtained by the peak area ratios (each peak was divided by an IS peak area) and concentrations. For quantification of GABA, caffeic acid, and ferulic acid in *L*. *paucicostata*, the same concentration of IS (2,000 mg/L myristic-*d*_*27*_ acid) was used. The conditions used for quantitative analysis were as previously reported [23], after modification of oven temperature settings. The initial oven temperature was set at 100°C and programmed to increase up to 150°C (at 5°C/min) and then to 180°C (at 3°C/min), then to 185°C (at 5°C/min and holding for 5 min), then to 200°C (at 3°C/ min), then to 225°C (at 5°C/min), then to 230°C (at 3°C/min). GC-MS analyses of GABA, caffeic acid, and ferulic acid were performed in triplicate. The quantification of GABA, caffeic acid, and ferulic acid was accomplished using the regression equations listed in S1 Table.

### Statistical analyses

SPSS Statistics 25 software (IBM, Somers, Conn., USA) was used to perform statistical analyses, and significant differences were assessed using a Mann-Whitney test and Student’s *t*-test (*p* < 0.05). Principal component analysis (PCA) was performed using the SIMCA software (version 15.0.2, Sartorius Stedim Data Analytics AB, Umeå, Sweden), with mean-centered and unit-variance scaling pretreatment.

## Results and discussion

### Growth of *L*. *paucicostata* cultures under ethephon treatment

**Fig 1:**
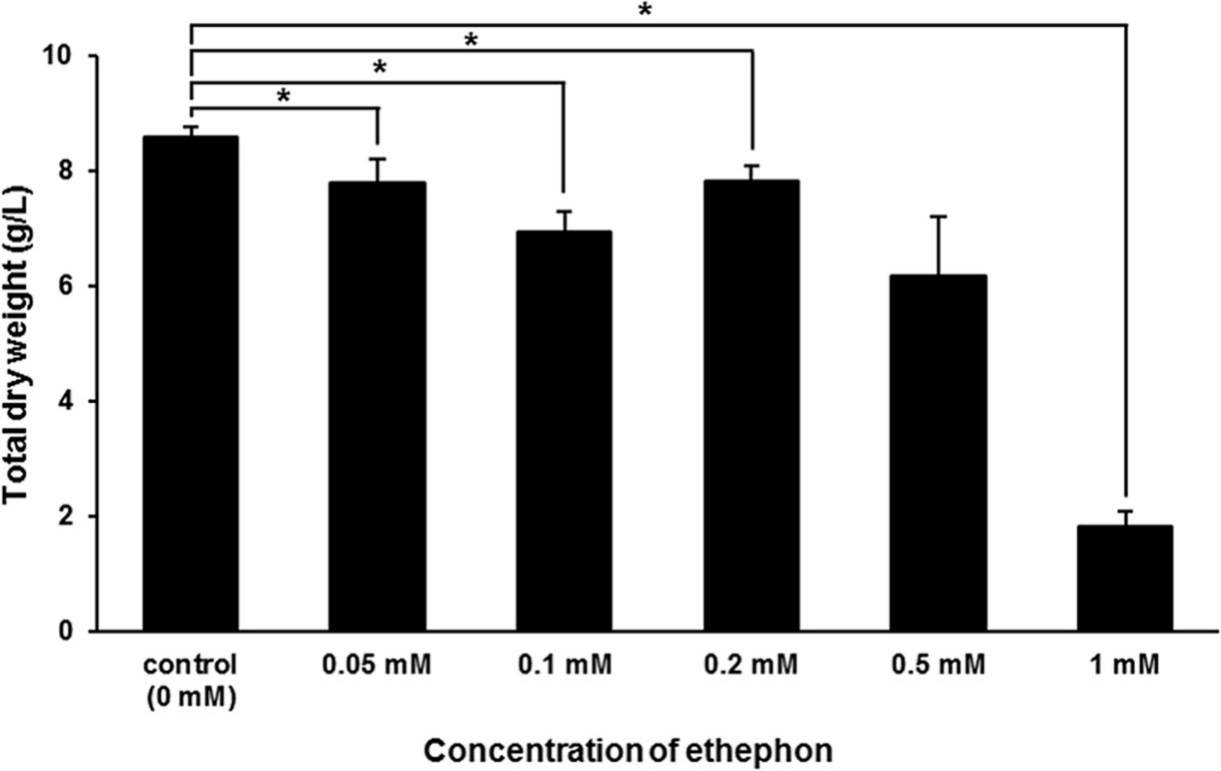
Effects of various ethephon concentrations on total dry weight of *L*. *paucicostata*. Vertical bars indicate mean values and error bars represent the standard deviation (n = 3) of each treatment group. An asterisk indicates significant differences (*p* < 0.05) between control and treated groups based on Student´s *t*-test.

Whole plants of *L*. *paucicostata* were exposed to various concentrations of ethephon (0 [control], 0.05, 0.1, 0.2, 0.5, or 1 mM). An overall decrease in total DW was observed in all ethephon-treated groups compared to the control group (Fig 1); further, total DW decreased significantly in all ethephon-treated groups, except for the 0.5 mM treatment group. Additionally, we observed that the total number of fronds of *L*. *paucicostata* increased under 0.05 and 0.1 mM ethephon treatment, whereas it decreased under 0.2, 0.5, and 1 mM ethephon treatment (S2 Fig).

Depending on plant species, tissue type, and ethylene concentration, ethylene affects growth, number, or plant size differently [27]. Thus, for example, in some hydrophytes such as rice, ethylene accelerates growth, whereas in others, such as *Lemna minor*, it hampers growth [7, 28]. A previous study showed that ethephon treatment increased the total number of corms without changing the total weight of potted Freesia [13]. In turn, ethephon treatment reduced the size of leaf blades in several plants, including *Avena sativa*, *Agrostis* sp., and *Poa pratensis* [29]. Similarly, we observed an increased number of fronds and reduced total DW under ethephon treatment in *L*. *paucicostata* cultures.

### Ethephon-induced alteration of metabolic profiles in *L*. *paucicostata* cultures

**Fig 2:**
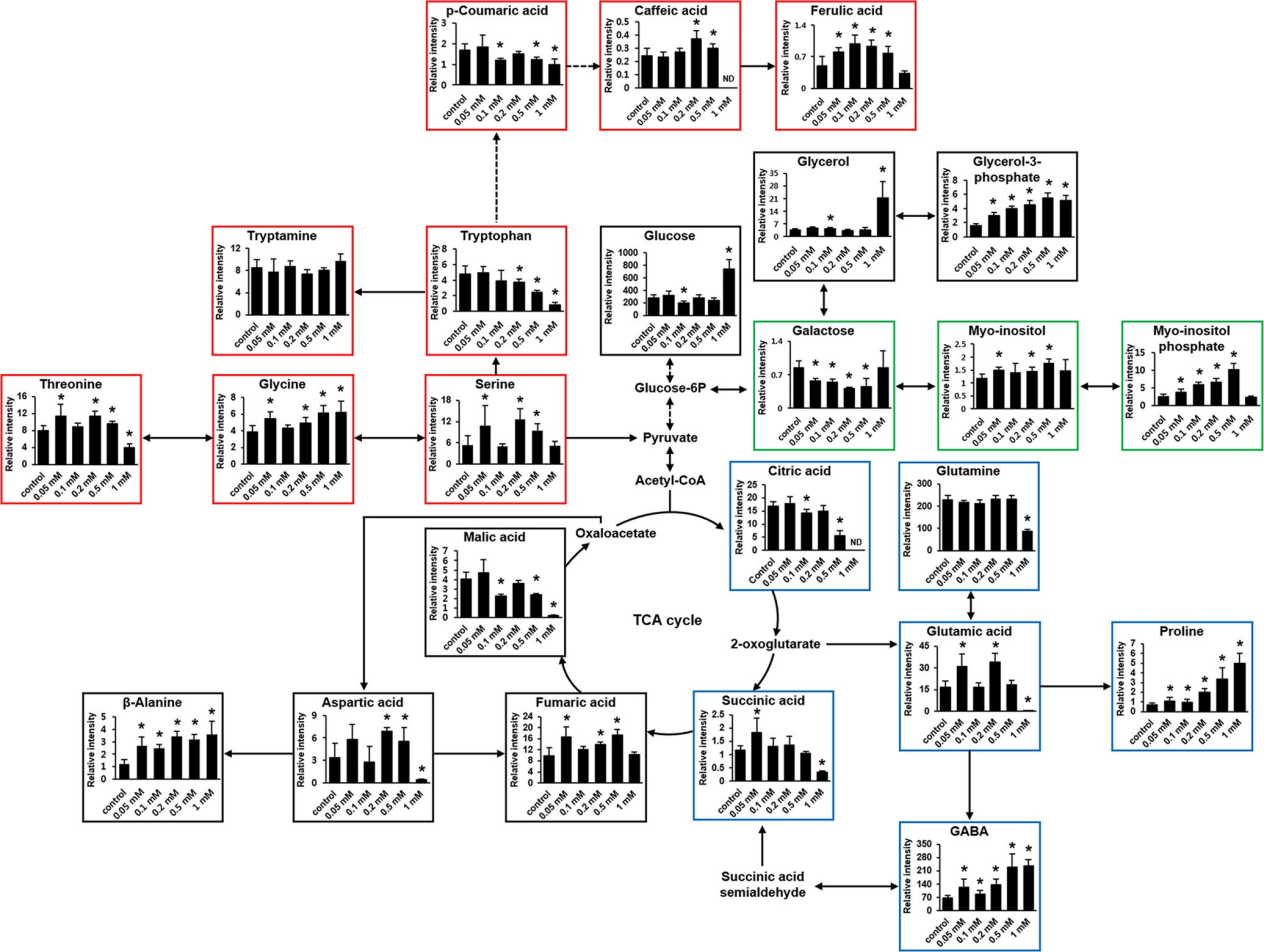
Relative levels of major metabolites of *L*. *paucicostata* cultivated under various ethephon concentrations at day 35. The suggested pathway of metabolites was expressed in accordance with the KEGG database (http://www.genome.jp/kegg/). Data are means and error bars indicate SD for nine measurements (n = 9, 3 biological replicates and 3 technical replicates). Mann-Whitney test (*p* < 0.05) was conducted to obtain significant differences between the control and ethephon-treated groups. Significant differences for each ethephon treatment group are indicated by an asterisk.

Forty-eight metabolites were detected in *L*. *paucicostata* by GC-MS (Table 1). Significant differences in relative intensity were observed among the metabolites in the different treatment groups (Fig 2, S2 Table). Ethephon treatment significantly increased relative levels of most alcohols in almost all treatment groups; however, the relative levels of amino acids significantly increased under treatment with ethephon at 0.05, 0.2, and 0.5 mM, but significantly decreased under 1 mM ethephon. Conversely, no changes were observed under 0.1 mM ethephon. Most fatty acids significantly increased in the ethephon-treated groups; organic acids exhibited a significantly increasing trend under 0.05 and 0.2 mM ethephon treatments but a significantly decreasing trend after treatment with 0.1, 0.5, and 1 mM ethephon. Overall, ethephon increased the phenolic compounds content and decreased the sugar content. Interestingly, all concentrations of ethephon significantly increased the GABA content (Fig 2, S2 Table).

**Table 1:** Peak assignment and identification of various metabolites in *L*. *paucicostata* obtained by GC-MS analysis.

NO.	Compound	RT	TMS	Ion fragment (m/z)
	**Alcohols**			
1	Glycerol	13.40	3	103, 117, **205**, 299
2	Glycerol-3-phosphate	27.95	4	103, 299, **357**, 445
3	Myo-inositol	39.28	6	191, 217, **305**, 318
4	Myo-inositol phosphate	47.53	7	217, 299, **318**, 387
	**Amino acids**			
5	Alanine	8.80	2	100, **116**, 133, 190
6	β-Alanine	17.26	3	86, **174**, 248, 290
7	Asparagine	22.21	2	100, 116, 130, **159**
		24.77	3	**116**, 132, 188, 231
8	Aspartic acid	19.77	3	100, 202, 218, **232**
9	Cysteine	20.84	3	100, 132, **220**, 294
10	Glutamic acid	23.10	3	128, 156, 218, **246**
11	Glutamine	28.26	3	128, **156**, 245, 347
12	Glycine	14.17	3	86, 133, **174**, 248
13	Histidine	32.88	3	**154**, 218, 254, 356
14	Isoleucine	13.85	3	**158**, 180, 218, 299
15	Lysine	33.16	4	128, 156, 230, **317**
16	Proline	13.94	2	99, 133, **142**, 216
17	Pyroglutamic acid	19.64	2	**156**, 230, 258, 273
18	Serine	12.90	2	57, 103, **116**, 132
		15.66	3	100, 188, **204**, 218
19	Threonine	16.30	3	101, 117, **218**, 291
20	Tryptophan	42.78	3	100, **202**, 218, 291
21	Valine	8.44	1	55, **72**, 146, 156
		11.79	2	100, **144**, 145, 218
	**Fatty acids**			
22	Glycerol monostearate	54.12	2	57, 129, 205, **399**
23	Linoleic acid	42.95	1	81, 117, 129, **337**
24	α-Linoleic acid	43.10	1	**79,** 95, 108, 129
25	Palmitic acid	37.98	1	**117**, 132, 145, 313
26	Stearic acid	43.84	1	**117**, 129, 132, 341
	**Organic acids**			
27	Citric acid	29.82	4	**273**, 347, 363, 375
28	Erythronic acid	20.49	4	117, 205, 220, **292**
29	Fumaric acid	15.45	2	115, 133, 143, **245**
30	Glyceric acid	14.87	3	103, 133, **189**, 292
31	3-Hydroxy-3-methyl glutaric acid	22.44	3	115, 231, **247**, 273
32	2-Keto-D-gluconic acid	18.56	4	103, 117, 205, **234**
33	Malic acid	18.83	3	101, 175, 190, **233**
34	Succinic acid	14.47	2	55, 129, 172, **247**
	**Phenolics**			
35	Caffeic acid	40.93	3	191, **219**, 381, 396
36	p-Coumaric acid	33.70	2	**218**, 249, 293, 308
37	Ferulic acid	39.41	2	249, 308, 323, **338**
	**Sugars**			
38	Fructose	29.29	5	129, **217**, 257, 437
		31.48	5 (MEOX)	**103**, 133, 217, 307
39	Galactose	32.47	5	129, 191, **204**, 217
40	Glucose	32.11	5	129, 191, **204**, 217
		32.27	5 (MEOX)	160, 205, 217, **319**
41	Sucrose	51.81	8	217, 271, **361**, 437
	**Others**			
42	γ-Aminobutyric acid (GABA)	20.01	3	86, **174**, 216, 304
43	Phosphoric acid	13.28	3	133, 211, **299**, 314
44	Serotonine	48.46	4	86, **174**, 290, 449
45	Suberylglycine	23.30	3	**188**, 216, 231, 303
46	Threonic acid	20.50	4	117, 205, 220, **292**
47	Threonic acid-1,4-lactone	15.92	2	101, 116, 131, **247**
48	Tryptamine	42.90	3	86, 100, **174**, 361

Base peak of each compound is represented with bold characters. RT, retention time; TMS, trimethylsilylation; MEOX, methoxylamine hydrochloride.

The relative level of galactose decreased, while the relative levels of myo-inositol and myo-inositol phosphate increased in most ethephon-treated groups (Fig 2, green boxes). We hypothesized that galactose was used to synthesize myo-inositol and myo-inositol phosphate under ethephon treatment, whereby its content decreased. Myo-inositol plays a key role in growth and development, especially in *Acer* and *Lemna* species [30]. In turn, inositol phosphate plays a role in cell signaling in *Arabidopsis thaliana* [31]. Wang et al [32] reported that ethylene treatment of the cytoplasm of rubber tree cells induced an increase in myo-inositol phosphate synthase, which converts glucose-6-phosphate into myo-inositol phosphate. We speculated that myo-inositol phosphate synthase might be induced by ethephon treatment and consequently, the relative levels of myo-inositol and myo-inositol phosphate increased.

Proline and GABA levels increased in all ethephon-treated groups without alteration in glutamine levels (except at 1 mM ethephon). The level of glutamic acid increased under ethephon treatment at 0.05 mM and 0.2 mM and decreased at 1 mM (blue boxes in Fig 2). It is likely that glutamic acid was utilized to produce proline and GABA, but not glutamine. Proline and GABA are reportedly regulators of stress tolerance [33]. A previous study on olive leaves showed that ethephon treatment increased reactive oxygen species (ROS) production resulting in oxidative stress [34]. Furthermore, previous studies have shown that exogenous ethephon treatment induced an increase in GABA levels to resist oxidative stress in *Chlorella vulgaris* [27]. Especially, GABA is synthesized from glutamic acid through glutamic acid decarboxylase, and this enzyme regulated by Ca^2+^/calmodulin (CaM) accumulation [35]. Previous studies have shown that environmental stress increased cellular levels of Ca^2+^ [36, 37]. The glutamic acid decarboxylase of most plants has calmodulin-binding domains (CaM-BDs) [35] that bind calmodulin in the presence of Ca^2+^ [38, 39]. Thus, we believe that the increased level of GABA might be the result from oxidative stress induced by ethephon treatment, and it might be caused by increased activity of glutamic acid decarboxylase.

The relative levels of serine, glycine, and threonine increased in ethephon-treated groups. Similarly, the levels of caffeic acid and ferulic acid also increased in most ethephon-treated groups, except for the group treated with 1 mM ethephon. However, the level of tryptophan and p-coumaric acid decreased in most treatment groups, whereas the level of tryptamine showed no change. Serine is the precursor of glycine and tryptophan biosynthesis. Tryptophan can be used for biosynthesis of p-coumaric acid. Thus, p-coumaric acid may be converted to caffeic and ferulic acids (red boxes in Fig 2). Ke et al. [40] reported that ethylene treated-lettuce showed an approximately 9-fold increase in the concentration of caffeic acid relative to the control group. Similarly, ferulic acid production in carrots also reportedly increased upon ethylene treatment [41]. Therefore, we believe that ethephon might play a role in converting serine to tryptophan, and subsequently tryptophan to caffeic and ferulic acids, via p-coumaric acid.

**Fig 3:**
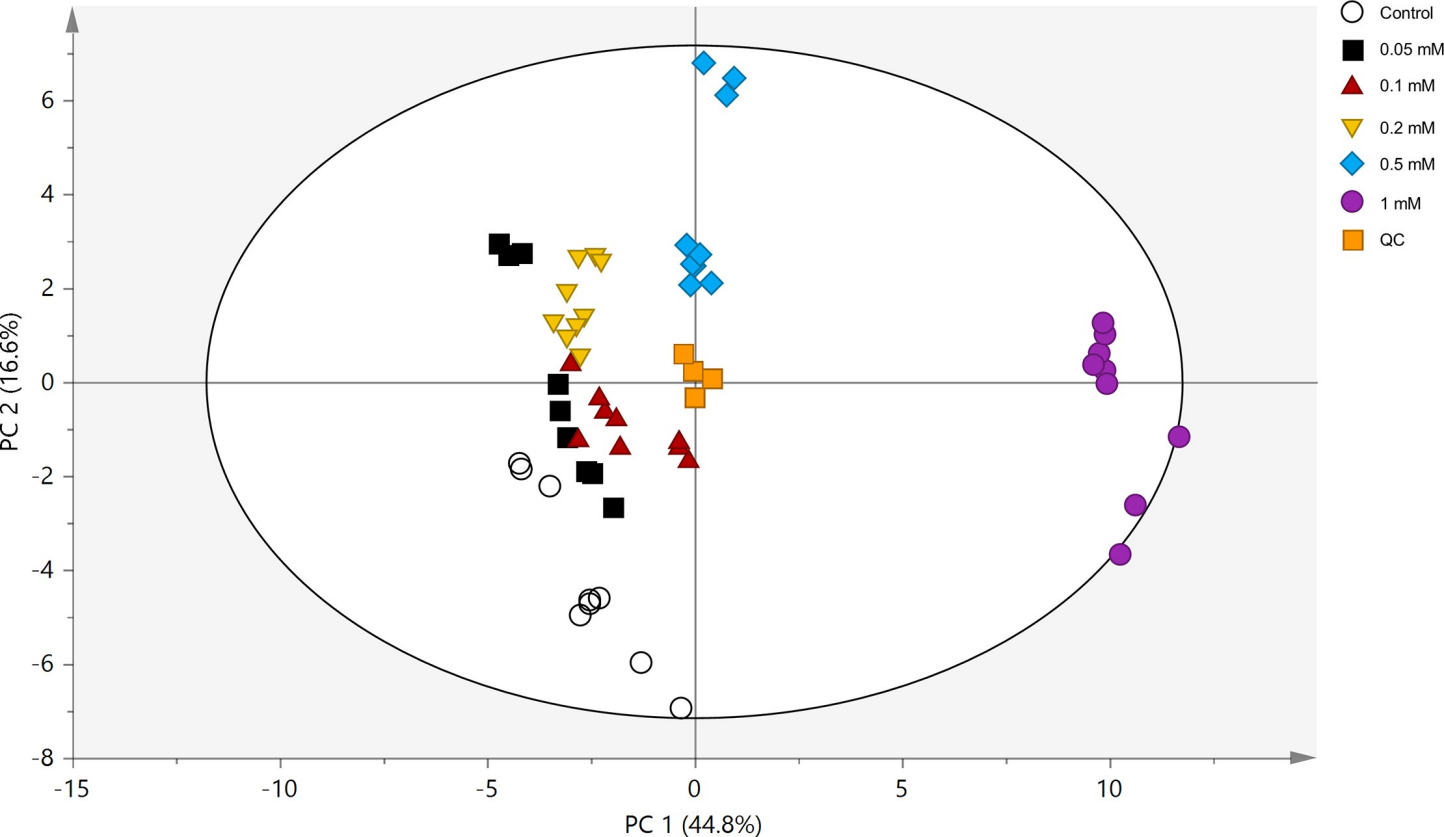
PCA-derived score plots including QC samples. Treatment with 0.05, 0.1, 0.2, 0.5, and 1 mM ethephon and quality control (QC), are represented by ○, ■ (black), ▲, ▼, ◆, ●, and ■ (orange), respectively.

The metabolic profile obtained by GC-MS was plotted with the PCA-derived score plot. The PCA-derived score plot of *L*. *paucicostata* and quality control (QC) samples was used to explore interrelations between treatment groups (Fig 3). A high aggregation of all QC samples in GC-MS was detected in the PCA-derived score plot, suggesting high stability of the GC-MS analytical platform during the entire experiment. Treatment groups were distinctly separated, whereas samples clustered tightly together. As ethephon concentration increased, the separation between control and treatment groups increased. This indicates that ethephon treatment greatly influenced the metabolic profiles of *L*. *paucicostata*.

Based on the altered metabolic profiles induced by ethephon treatment, we focused on caffeic acid, GABA, and ferulic acid, which showed increased relative intensity levels in unit cells and are known to be beneficial for human health and disease control [42–47]. We quantified these compounds to obtain an estimate of their production by *L*. *paucicostata* culture.

### Production of GABA and ferulic acid in *L*. *paucicostata* cultures under various concentrations of exogenous ethephon

Although the relative level of caffeic acid in unit cells increased (Fig 2), there was a significant decrease in caffeic acid production by ethephon treatment (Table 2). Therefore, we focused on the production of GABA and ferulic acid.

**Table 2:** Production of GABA, caffeic acid, and ferulic acid in *L*. *paucicostata* culture under various concentrations of ethephon.

Compounds (mg/L)	Control	0.05 mM	0.1 mM	0.2 mM	0.5 mM	1 mM
γ-Aminobutyric acid (GABA)	1.655 ± 0.551	3.101 ± 0.808*	2.296 ± 0.789	3.394 ± 0.895*	5.041 ± 1.373*	1.599 ± 0.589
Caffeic acid	0.270 ± 0.003	0.255 ± 0.013*	0.233 ± 0.013*	0.270 ± 0.009	0.225 ± 0.036*	ND
Ferulic acid	0.432 ± 0.077	0.592 ± 0.033*	0.600 ± 0.084*	0.640 ± 0.071*	0.571 ± 0.024*	0.129 ± 0.037*

Data are means ± SD for nine measurements (three biological and experimental replicates).

* denotes significant differences (*p* < 0.05) between control and ethephon treatment groups (0.05, 0.1, 0.2, 0.5, and 1 mM) on day 35, based on Mann-Whitney test. ND, not detected.

The production of GABA and ferulic acid ranged from 1.599 to 5.041 and from 0.129 to 0.640 mg/L, respectively (Table 2). The highest production of GABA (5.041 ± 1.373 mg /L) was observed in the 0.5 mM ethephon-treated group, followed by 0.2 mM (3.394 ± 0.895 mg/L) and 0.05 mM ethephon-treated (3.101 ± 0.808 mg/L) groups (Table 2). In animals, GABA is a known neurotransmitter of the central nervous system, while in plants it acts in cell signaling and accumulates in response to environmental stress [33, 48]. It is also used as a relaxer and hormone regulator and in the treatment of insomnia, narcolepsy, and epilepsy [42–44, 46]. Our results showed that 0.5 mM ethephon was the optimum concentration to increase GABA production, whereas 1 mM ethephon did not increase GABA production.

Ferulic acid is a natural phenol that possibly plays a role in antioxidation. In addition, Park et al. [47] found that ferulic acid extracted from *Tetragonia tetragonioides* showed whitening and anti-wrinkle properties [47]. In combination with ascorbic acid and α-tocopherol, ferulic acid induces the retardation of thymine dimer formation and ROS in the epidermis [45]. The highest production of ferulic acid (0.640 ± 0.071 mg/L) was observed in the 0.2 mM ethephon treatment (Table 2). Except for the 1 mM treatment, ferulic acid production significantly increased in all treatment groups. Therefore, treatment of *L*. *paucicostata* cultures with 0.2 mM ethephon could be used to enhance the production of ferulic acid, for use in the cosmetic and pharmaceutical industries.

## Conclusions

In this study, we investigated the effect of ethephon on the growth and metabolic profiles of *L*. *paucicostata* cultures. This is the first study to observe the effect of various ethephon concentrations on the growth and the comprehensive metabolic profiles of *L*. *paucicostata*. Total weight of *L*. *paucicostata* significantly decreased under all treatments tested. Overall, ethephon treatment increased the relative levels of alcohols, amino acids, fatty acids, and phenolic compounds, but decreased organic acids and sugars. Interestingly, the highest productions of GABA and ferulic acid were observed in the 0.5 and 0.2 mM ethephon treatment groups, respectively. Our results could be applied for large-scale cultivation of *L*. *paucicostata* with enhanced content of GABA and ferulic acid and could benefit various industrial purposes.

## Supporting information

S1 Fig Photographs of *L*. *paucicostata* culture under various ethephon concentrations on days 7, 14, 21, 28, and 35. (TIF)

S2 Fig Total number fronds of *L*. *paucicostata* cultivated under various concentrations of ethephon treatment. Data represent the mean values, and the vertical bars indicate the standard deviation from three biological replications.
(TIF)

S1 Table Regression equation, R^2^, LOD, and LOQ of GABA, caffeic acid, and ferulic acid standard. (DOCX)

S2 Table Relative levels of various metabolites of *L. paucicostata* culture under various concentrations of ethephon treatment analyzed by GC-MS. Date are mean ± SD values for 9 measurements (biological three replicate and experimental three replicate). The relative levels of metabolites were obtained by dividing each peak intensity by the internal standard peak intensity (myristic acid-*d_27_*). Those were multiplied by 100 and presented in this table. The significant differences between control and ethephon-treated groups were indicated as * and this was made with Mann-Whiteny test (*p* < 0.05).
(DOCX)
