# Supplementary figures and images for: Alteration of metabolic profiles in Lemna paucicostata culture and enhanced production of GABA and ferulic acid by ethephon treatment
Source: PLoS One. 2020 Apr 16;15(4):e0231652. doi: 10.1371/journal.pone.0231652 (PMC7162458; doi:10.1371/journal.pone.0231652)

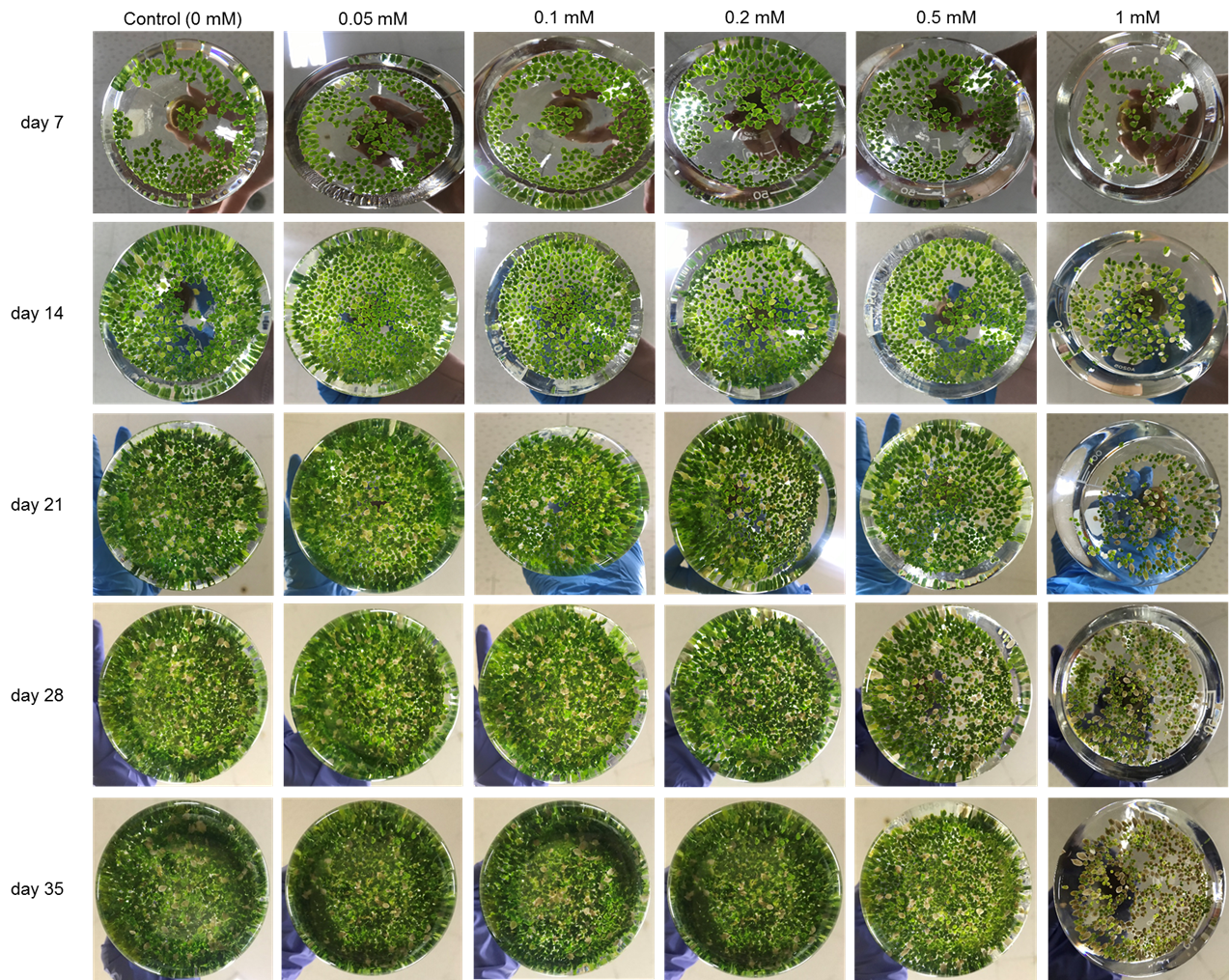

Supplement: S1 Fig — (TIF) [file pone.0231652.s001.tif]

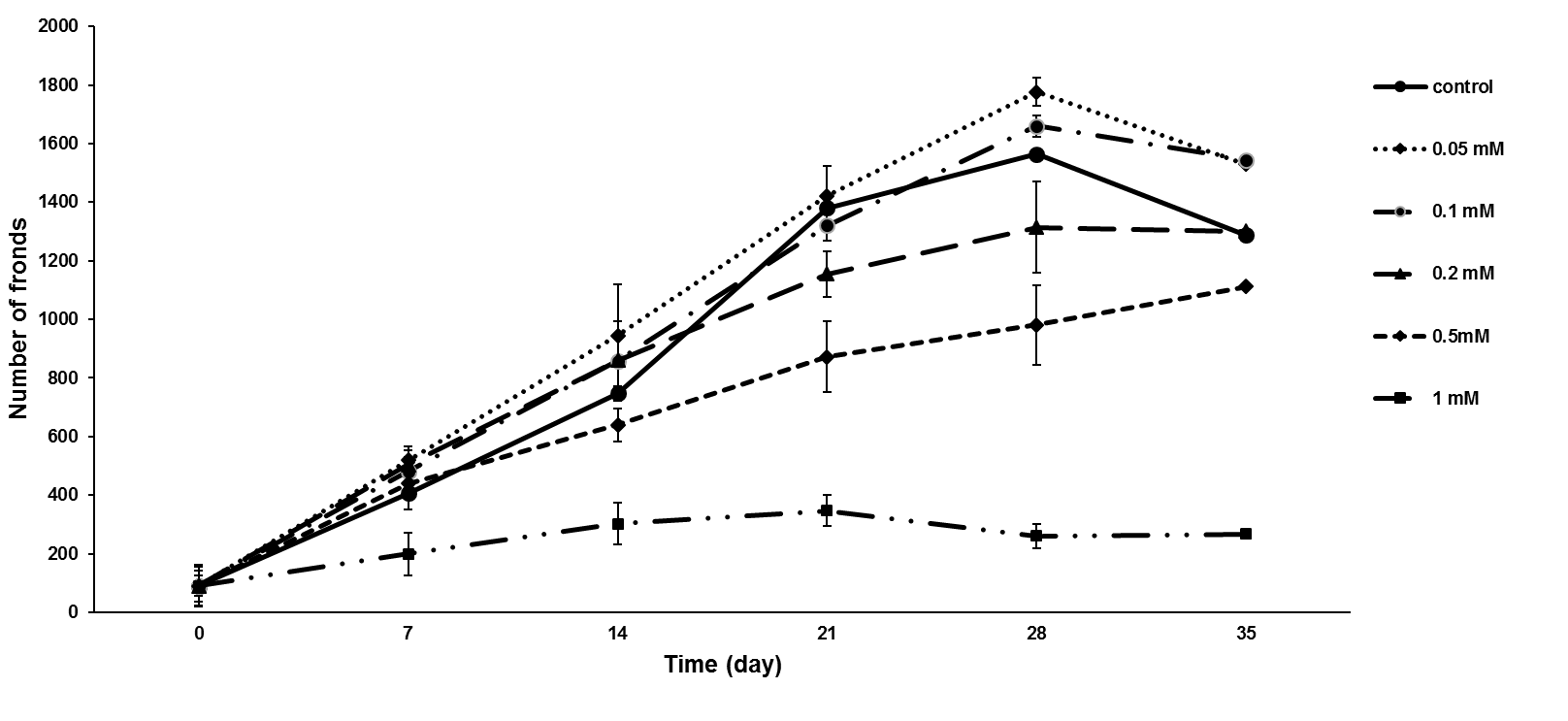

Supplement: S2 Fig — Data represent the mean values, and the vertical bars indicate the standard deviation from three biological replications. (TIF) [file pone.0231652.s002.tif]
