# Supplementary material for: Alteration of metabolic profiles in Lemna paucicostata culture and enhanced production of GABA and ferulic acid by ethephon treatment
Source: PLoS One. 2020 Apr 16;15(4):e0231652. doi: 10.1371/journal.pone.0231652 (PMC7162458; doi:10.1371/journal.pone.0231652)
Supplement: S1 Table — (DOCX) [file pone.0231652.s003.docx]

**S1 Table. Regression equation, R^2^, LOD, and LOQ of GABA, caffeic acid, and ferulic acid standard.**

| **Compound** | **regression equation** | **R^2^ values** | **LOD (μg/mL)** | **LOQ (μg/mL)** |
| --- | --- | --- | --- | --- |
| γ-Aminobutyric acid (GABA) | y = 0.0293x - 0.0065 | 0.997 | 0.64 | 1.93 |
| Caffeic acid | y = 0.0164x - 0.0209 | 0.983 | 0.05 | 0.15 |
| Ferulic acid | y = 0.0053x - 0.0068 | 0.976 | 0.22 | 0.67 |

LOD, Limit of detection; LOQ, Limit of quantitation.
