# Supplementary material for: Alteration of metabolic profiles in Lemna paucicostata culture and enhanced production of GABA and ferulic acid by ethephon treatment
Source: PLoS One. 2020 Apr 16;15(4):e0231652. doi: 10.1371/journal.pone.0231652 (PMC7162458; doi:10.1371/journal.pone.0231652)
Supplement: S2 Table — Date are mean ± SD values for 9 measurements (biological three replicate and experimental three replicate). The relative levels of metabolites were obtained by dividing each peak intensity by the internal standard peak intensity (myristic acid-d27). Those were multiplied by 100 and presented in this table. The significant differences between control and ethephon-treated groups were indicated as * and this was made with Mann-Whiteny test (p < 0.05). (DOCX) [file pone.0231652.s004.docx]

**S2 Table. Relative levels of various metabolites of *L. paucicostata* culture under various concentrations of ethephon treatment analyzed by GC-MS.**

| **NO.** | **Compound** | **Control** | **0.05 mM** | **0.1 mM** | **0.2 mM** | **0.5 mM** | **1 mM** |
| --- | --- | --- | --- | --- | --- | --- | --- |
|  | **Alcohols** |  |  |  |  |  |  |
| 1 | Glycerol | 3.47 ± 0.72 | 4.58 ± 0.88 | 4.49 ± 0.37* | 3.18 ± 0.72 | 3.76 ± 1.31 | 21.39 ± 9.00* |
| 2 | Glycerol-3-phosphate | 1.55 ± 0.30 | 3.00 ± 0.43* | 3.96 ± 0.39* | 4.51 ± 0.64* | 5.51 ± 0.70* | 5.13 ± 0.68* |
| 3 | Myo-inositol | 1.17 ± 0.18 | 1.48 ± 0.14* | 1.38 ± 0.37 | 1.44 ± 0.18* | 1.76 ± 0.16* | 1.47 ± 0.42 |
| 4 | Myo-inositol phosphate | 2.51 ± 0.63 | 3.78 ± 0.74* | 5.94 ± 0.68* | 6.61 ± 1.07* | 10.18 ± 1.72* | 2.28 ± 0.42 |
|  | **Amino acids** |  |  |  |  |  |  |
| 5 | Alanine | 19.82 ± 5.20 | 22.15 ± 4.01 | 15.53 ± 4.31 | 23.97 ± 6.21 | 29.02 ± 12.02 | 36.21 ± 21.16 |
| 6 | β-Alanine | 1.21 ± 0.35 | 2.66 ± 0.75* | 2.47 ± 0.30* | 3.43 ± 0.39* | 3.17 ± 0.43* | 3.61 ± 1.06* |
| 7 | Asparagine | 125.41 ± 18.93 | 109.92 ± 8.78 | 103.85 ± 7.30* | 106.28 ± 12.60* | 96.15 ± 13.40* | 0.17 ± 0.02* |
| 8 | Aspartic acid | 3.38 ± 1.89 | 5.82 ± 1.96 | 2.79 ± 2.06 | 6.85 ± 0.55* | 5.57 ± 1.82* | 0.44 ± 0.10* |
| 9 | Cysteine | 6.15 ± 1.43 | 3.59 ± 1.64* | 5.99 ± 2.43 | 4.76 ± 2.76 | 5.78 ± 1.72 | 1.40 ± 0.11* |
| 10 | Glutamic acid | 16.64 ± 4.19 | 30.99 ± 8.61* | 16.47 ± 3.11 | 33.79 ± 6.33* | 18.56 ± 3.01 | 0.21 ± 0.06* |
| 11 | Glutamine | 229.77 ± 18.65 | 218.59 ± 7.87 | 211.77 ± 17.72 | 231.31 ± 17.66 | 233.15 ± 15.80 | 85.92 ± 8.97* |
| 12 | Glycine | 3.88 ± 0.74 | 5.44 ± 0.85* | 4.30 ± 0.42 | 4.90 ± 0.68* | 6.16 ± 0.92* | 6.24 ± 1.30* |
| 13 | Histidine | 6.00 ± 1.28 | 5.51 ± 0.68 | 4.41 ± 1.12* | 4.38 ± 0.50* | 5.22 ± 0.68 | ND |
| 14 | Isoleucine | 9.03 ± 2.18 | 12.02 ± 3.67 | 8.00 ± 2.07 | 10.09 ± 0.62 | 7.48 ± 1.04 | 13.56 ± 2.28* |
| 15 | Lysine | 0.58 ± 0.08 | 0.77 ± 0.19 | 0.64 ± 0.09 | 0.61 ± 0.10 | 0.30 ± 0.04* | ND |
| 16 | Proline | 0.67 ± 0.19 | 1.09 ± 0.39* | 0.98 ± 0.32* | 2.02 ± 0.38* | 3.34 ± 1.22* | 4.97 ± 1.04* |
| 17 | Pyroglutamic acid | 99.97 ± 7.12 | 87.11 ± 9.48* | 78.62 ± 16.65* | 95.27 ± 13.34 | 112.54 ± 10.97* | 40.24 ± 2.72* |
| 18 | Serine | 5.29 ± 2.72 | 10.82 ± 5.67* | 4.97 ± 0.83 | 12.60 ± 2.98* | 9.42 ± 1.96* | 5.26 ± 1.16 |
| 19 | Threonine | 7.95 ± 1.28 | 11.41 ± 2.82* | 8.94 ± 0.84 | 11.38 ± 1.16* | 9.66 ± 0.60* | 3.91 ± 0.94* |
| 20 | Tryptophan | 4.79 ± 1.01 | 4.90 ± 0.82 | 3.86 ± 1.40 | 3.74 ± 0.42* | 2.42 ± 0.24* | 0.84 ± 0.30* |
| 21 | Valine | 23.28 ± 4.66 | 26.41 ± 3.41 | 22.76 ± 3.57 | 26.39 ± 3.04 | 33.43 ± 4.77* | 30.75 ± 3.60* |
|  | **Fatty acids** |  |  |  |  |  |  |
| 22 | Glycerol monostearate | 4.85 ± 1.37 | 7.76 ± 1.20* | 8.65 ± 1.28* | 7.79 ± 2.74* | 7.21 ± 1.55* | 5.54 ± 1.76 |
| 23 | Linoleic acid | 0.57 ± 0.15 | 0.55 ± 0.09 | 0.83 ± 0.08* | 0.80 ± 0.06* | 0.72 ± 0.05* | 0.70 ± 0.28 |
| 24 | α-Linoleic acid | 2.66 ± 0.78 | 3.08 ± 0.49 | 4.79 ± 0.74* | 4.21 ± 0.37* | 3.41 ± 0.45* | 2.00 ± 1.22 |
| 25 | Palmitic acid | 3.31 ± 0.58 | 3.38 ± 0.52 | 4.47 ± 0.30* | 3.97 ± 0.26* | 4.35 ± 0.38* | 18.97 ± 2.46* |
| 26 | Stearic acid | 0.61 ± 0.14 | 0.63 ± 0.08 | 0.79 ± 0.10* | 0.76 ± 0.10* | 0.77 ± 0.12* | 2.53 ± 0.31* |
|  | **Organic acids** |  |  |  |  |  |  |
| 27 | Citric acid | 16.89 ± 1.69 | 17.86 ± 2.61 | 14.33 ± 1.35* | 14.94 ± 2.19 | 5.56 ± 1.94* | ND |
| 28 | Erythronic acid | 0.35 ± 0.06 | 0.40 ± 0.05 | 0.31 ± 0.05 | 0.41 ± 0.04* | 0.34 ± 0.04 | 0.26 ± 0.04* |
| 29 | Fumaric acid | 9.94 ± 2.90 | 16.70 ± 3.47* | 12.16 ± 1.02 | 13.87 ± 1.00* | 17.38 ± 1.89* | 10.34 ± 0.88 |
| 30 | Glyceric acid | 0.69 ± 0.11 | 0.82 ± 0.09* | 0.61 ± 0.14 | 0.64 ± 0.13 | 0.43 ± 0.05* | 0.17 ± 0.02* |
| 31 | 3-Hydroxy-3-methyl glutaric acid | 13.19 ± 4.10 | 11.68 ± 4.13 | 4.06 ± 1.27* | 6.88 ± 0.98* | 2.63 ± 1.29* | 0.38 ± 0.23* |
| 32 | 2-Keto-D-gluconic acid | 0.51 ± 0.08 | 0.67 ± 0.10* | 0.69 ± 0.13* | 0.72 ± 0.20* | 0.78 ± 0.27* | 0.27 ± 0.08* |
| 33 | Malic acid | 4.06 ± 0.69 | 4.69 ± 1.42 | 2.24 ± 0.22* | 3.55 ± 0.38 | 2.38 ± 0.16* | 0.23 ± 0.04* |
| 34 | Succinic acid | 1.16 ± 0.17 | 1.84 ± 0.54* | 1.31 ± 0.31 | 1.37 ± 0.32 | 1.05 ± 0.08 | 0.35 ± 0.03* |
|  | **Phenolics** |  |  |  |  |  |  |
| 35 | Caffeic acid | 0.24 ± 0.06 | 0.23 ± 0.04 | 0.27 ± 0.03 | 0.37 ± 0.06* | 0.30 ± 0.03* | ND |
| 36 | p-Coumaric acid | 1.68 ± 0.31 | 1.84 ± 0.57 | 1.19 ± 0.11* | 1.50 ± 0.14 | 1.22 ± 0.14* | 0.98 ± 0.28* |
| 37 | Ferulic acid | 0.50 ± 0.20 | 0.80 ± 0.10* | 0.98 ± 0.19* | 0.93 ± 0.13* | 0.78 ± 0.14* | 0.33 ± 0.05 |
|  | **Sugars** |  |  |  |  |  |  |
| 38 | Fructose | 160.14 ± 26.13 | 156.28 ± 13.87 | 118.18 ± 17.49* | 149.31 ± 12.36 | 145.25 ± 23.11 | 197.05 ± 44.22* |
| 39 | Galactose | 0.84 ± 0.15 | 0.57 ± 0.05* | 0.54 ± 0.07* | 0.41 ± 0.03* | 0.45 ± 0.17* | 0.84 ± 0.36 |
| 40 | Glucose | 284.90 ± 41.32 | 320.23 ± 72.82 | 198.86 ± 28.87* | 277.29 ± 51.87 | 242.20 ± 34.96 | 745.75 ± 146.48* |
| 41 | Sucrose | 361.83 ± 53.90 | 336.15 ± 31.19 | 336.21 ± 23.40 | 340.81 ± 49.04 | 319.94 ± 28.18 | 297.29 ± 58.31* |
|  | **Others** |  |  |  |  |  |  |
| 42 | γ-Aminobutyric acid (GABA) | 67.46 ± 14.80 | 123.86 ± 43.57* | 89.16 ± 17.37* | 137.79 ± 29.05* | 230.14 ± 70.29* | 237.82 ± 33.13* |
| 43 | Phosphoric acid | 65.00 ± 6.56 | 80.25 ± 12.16* | 100.54 ± 7.87* | 97.59 ± 13.90* | 276.38 ± 54.82* | 219.9 ± 15.36* |
| 44 | Serotonine | 37.78 ± 3.66 | 34.83 ± 4.60 | 35.30 ± 5.33 | 37.64 ± 5.28 | 29.57 ± 4.17* | 1.86 ± 0.21* |
| 45 | Suberylglycine | 61.94 ± 7.70 | 43.90 ± 15.27* | 39.28 ± 5.02* | 41.39 ± 2.85* | 32.70 ± 5.07* | 2.09 ± 0.73* |
| 46 | Threonic acid | 3.06 ± 0.39 | 2.93 ± 0.22 | 2.90 ± 0.33 | 3.02 ± 0.44 | 2.21 ± 0.58* | 0.38 ± 0.20* |
| 47 | Threonic acid-1,4-lactone | 0.38 ± 0.05 | 0.51 ± 0.04* | 0.40 ± 0.03 | 0.41 ± 0.07 | 0.28 ± 0.03* | 0.17 ± 0.02* |
| 48 | Tryptamine | 8.53 ± 1.46 | 7.72 ± 2.36 | 8.70 ± 1.04 | 7.37 ± 0.77 | 8.02 ± 0.49 | 9.67 ± 1.36 |

Date are mean ± SD values for 9 measurements (biological three replicate and experimental three replicate). The relative levels of metabolites were obtained by dividing each peak intensity by the internal standard peak intensity (myristic acid-*d_27_*). Those were multiplied by 100 and presented in this table. The significant differences between control and ethephon treated groups were indicated as * and this was made with Mann-Whiteny test (*p* < 0.05).

ND, not detected.
